# Fracture Risk in Relation to Serum 25-Hydroxyvitamin D and Physical Activity: Results from the EPIC-Norfolk Cohort Study

**DOI:** 10.1371/journal.pone.0164160

**Published:** 2016-10-17

**Authors:** Cristina Julian, Marleen A. H. Lentjes, Inge Huybrechts, Robert Luben, Nick Wareham, Luis A. Moreno, Kay-Tee Khaw

**Affiliations:** 1 Department of Public Health and Primary Care, Institute of Public Health, School of Clinical Medicine, University of Cambridge, Cambridge, United Kingdom; 2 GENUD (Growth, Exercise, Nutrition and Development) Research Group, Instituto Agroalimentario de Aragón (IA2), Universidad de Zaragoza, Zaragoza, Spain; 3 Centro de Investigación Biomédica en Red de Fisiopatología de la Obesidad y Nutrición (CIBERObn), Universidad de Zaragoza, Zaragoza, Spain; 4 Dietary Exposure Assessment Group, International Agency for Research on Cancer, Lyon, France; 5 Department of Public Health and Primary Care, Institute of Public Health, Medical Research Council Epidemiology Unit, University of Cambridge, Cambridge, United Kingdom; University of Alabama at Birmingham, UNITED STATES

## Abstract

Vitamin D deficiency and physical inactivity have been associated with bone loss and fractures, but their combined effect has scarcely been studied either in younger or older adults. Therefore, we aimed to assess the associations between physical activity, age and 25-hydroxyvitamin D (25(OH)D) status separately and in combination with the incidence of fracture risk in the EPIC-Norfolk cohort study. Baseline (1993–1998) self-reported physical activity and serum 25(OH)D concentrations at follow-up (1998–2000) were collected in 14,624 men and women (aged 42–82 y between 1998 and 2000). Fracture incidence was ascertained up to March 2015. Cox proportional hazard model was used to determine HRs of fractures by plasma 25(OH)D (<30, 30 to <50, 50 to <70, 70 to <90, >90 nmol/L), age (<65 y and >65 y) and physical activity (inactive and active) categories, by follow-up time per 20 nmol/L increase in serum 25(OH)D and to explore age-25(OH)D and physical activity-25(OH)D interactions. The age-, sex-, and month-adjusted HRs (95% CIs) for all fractures (1183 fractures) by increasing vitamin D category were not significantly different. With additional adjustment for body mass index, smoking status, alcohol intake, supplement use and history of fractures, the fracture risk was 29% lower in those participants with 50 to 70 nmol/L compared with those in the lowest quintile (<30 nmol/L). Physical inactivity based on a single baseline assessment was not associated with fracture risk. Vitamin D status appeared inversely related to fractures in middle aged adults. In older adults, the relationship between vitamin D status and fracture risk was observed to be J-shaped. Clinical and public health practice in vitamin D supplementation could partially explain these findings, although definitive conclusions are difficult due to potential changes in exposure status over the long follow up period.

## Introduction

Worldwide, osteoporosis causes more than 8.9 million fractures annually, resulting in an osteoporotic fracture every 3 seconds [1]. In the UK, approximately 536,000 new fragility fractures appear each year and the economic burden of new and prior fractures is estimated to be £ 5,465 (€ 6,723) million per year by 2025 [2]. Multiple factors are known to influence fracture risk, such as falls, smoking, diet, physical activity (PA) and vitamin D status (25(OH)D) [3].

25(OH)D deficiency is a well-established cause of impaired bone mineralization that leads to osteomalacia in adults [3]. Findings of prospective cohort studies on the associations of 25(OH)D and fracture risk in older people are contradictory; while some show that higher 25(OH)D is associated with a lower risk of osteoporotic fractures [4–6], others do not demonstrate such association [7]. These inconsistent findings might be due to differences between study samples: inclusion of only postmenopausal women [7], adults over 65 years old [6] or low power small number of fracture cases [5–7].

There is considerable evidence from epidemiologic studies that physical inactivity is a risk factor for fractures [8]. A prospective study of more than 30,000 Danish men and women found that moderate levels of physical activity appear to provide protection against later hip fracture [9]. In the Nurses’ Health Study, postmenopausal women who reported walking at least 4 vs. 1 h/wk had a 41% lower risk of hip fracture [10].

There are no large-scale cohort studies on the combined effects of 25(OH)D status and PA on fracture risk. Only randomized clinical trials of the combined effect of physical fitness (strength, balance and mobility) and vitamin D supplementation on the prevention of fractures have been performed, but they have shown inconclusive results [11–13], partly because of differences in physical fitness protocols and supplement doses.

Therefore, the purpose of this study was to assess 25(OH)D in association with fracture risk separately and explore interactions with age and physical activity in a population-based cohort study.

## Methods

### Study design and population

The European Prospective Investigation into Cancer and Nutrition (EPIC)-Norfolk cohort analysed in this study is part of EPIC, a collaboration involving 10 European countries developed primarily to examine the association between diet and cancer, with additional health outcomes examined in EPIC-Norfolk. This cohort has been described in detail previously [14], but in brief, the Norfolk cohort consisted of 25,639 men and women aged 40–79 y living in the general community who participated in a baseline health examination (HE1) between 1993 and 1997 and a subsequent assessment (HE2) among 15,786 between 1997 and 2000. Both assessments were proceeded by a Health and Lifestyle Questionnaire (referred to as HLQ1 and HLQ2 respectively).

Eligible participants for the current analysis were 14,624 men and women who attended HE2 (aged 42–82 y at that time), with an available blood sample for 25(OH)D and who had data on physical activity from HLQ1.

The study was approved by the Norwich District Health Authority Ethics Committee, and all participants gave signed informed consent.

### Main exposure: Blood samples

Plasma and serum samples were obtained from venipuncture blood samples during HE2. Samples were stored in liquid nitrogen tanks until 2012 when serum samples were retrieved for 25(OH)D assays. Assays were conducted by VITAS, which is a reference laboratory in Nordic countries for fat soluble vitamins [15]. Assays for 25(OH)D were based on ultraperformance liquid chromatography interfaced by atmospheric pressure chemical ionization to mass spectrometry. This method measures the 25(OH)D_3_ and 25(OH)D_2_. The lower limit of detection was 1–4 nmol/L. The coefficients of variation (CV) for interassay analyses were 7.6% at 25(OH)D concentrations of 47.8 nmol/L and 6.9% at 83.0 nmol/L.

Only 923 individuals had measurable 25(OH)D_2_ (>1 nmol/L), the remainder were coded as zero. Population mean concentrations were 56.2 nmol/L (range 3.4–201.9 nmol/L) for 25(OH)D_3_ and 5.6 nmol/L (range 3.0–82.5 nmol/L) in those with detectable concentrations for 25(OH)D_2_. The total 25(OH)D concentration, was calculated as the sum of 25(OH)D_3_ and 25(OH)D_2_. We categorized total 25(OH)D into 5 categories by using clinically relevant cutoffs [16] as follows: <30, 30 to <50, 50 to <70, 70 to <90 and ≥90 nmol/L.

### Main exposure: Physical activity

Habitual PA was assessed from HLQ1 which included the short EPIC Physical Activity Questionnaire asking about PA during the preceding 12 months. The first section was about PA at work. The second section was about the amount of time spent in hours per week for summer and winter separately in each of the following activities: walking, cycling, gardening, do-it-yourself, physical exercise and housework. A simple PA-index was constructed to allocate participants to four ordered categories of overall activity [17]: inactive (sedentary job and no recreational activity); moderately inactive (sedentary job with <0.5 h recreational activity per day or standing job with no recreational activity); moderately active (sedentary job with 0.5–1 h recreational activity per day, or standing job with <0.5 h recreational activity per day, or physical job with no recreational activity); and active (sedentary job with >1 h recreational activity per day, or standing job with >0.5 h recreational activity per day, or physical job with at least some recreational activity, or heavy manual job). For the purpose of this study participants were categorized in two physical activity levels: physically inactive (inactive and moderately inactive) versus physically active (moderately active and active). The index was validated against a combined heart rate and movement sensor (with individual calibration) in 2000 participants from 10 European countries (representative of the EPIC-population with respect to age and sex). The pooled estimate of the correlation between self-reported and objectively assessed energy expenditure was r = 0.33[18]. We also showed a high repeatability of the index (weighted kappa = 0.6, p<0.001) [17].

### Ascertainment of incident fractures

Trained nosologists obtained vital status of the entire cohort based on death certificates of the United Kingdom Office of National Statistics. Individuals were also linked via their unique National Health Service number with the East Norfolk Health Authority (ENCORE) database, which identifies all hospital contacts throughout England and Wales for Norfolk residents [19]. International classification of diseases (ICD) 9 and 10 diagnostic codes were used to identify fracture events, representing all hip (120; S72), spine (225; S12,S22,S32,T08) and wrist (910; S52,S62) fracture cases in the cohort up to March 2015, an average of 15 ± 2.3 y of follow-up.

### Assessment of covariates

Trained nurses carried out a health examination. Height and weight were measured according to standard protocols [14], conducted at the participant’s general practitioner’s practice. Height was determined to the nearest millimetre by using a freestanding stadiometer. Weight was recorded to the nearest 0.2 kg with the participant wearing light clothing and no shoes. Body mass index (BMI) was estimated as weight divided by the square of height (kg/m^2^).

Participants also completed a self-administered health and lifestyle questionnaire (assessed at HLQ2). This included a self-reported medical history of diabetes, cancer, osteoporosis, arthritis and previous fractures; menopausal status, categorized as premenopausal, perimenopausal (<1 y), perimenopausal (1–5 y), or postmenopausal; and hormone replacement therapy (HRT) status; supplement use was ascertained by the question “Have you taken any vitamins, minerals, or other food supplements regularly during the past year (such as vitamin C, vitamin D, iron, calcium, fish oils, primrose oil, betacarotene, etc)?”, categorized as yes or no; smoking status was ascertained by asking “Have you ever smoked as much as one cigarette a day for as long as a year?” and “Do you smoke cigarettes now?”, categorized as current, former, or never users; and alcohol consumption was ascertained by asking about beer, wine and spirit consumption in a week (computed as units per week).

Participants’ occupation (assessed at HLQ1) was classified according to the Registrar General’s occupation-based classification scheme into 6 main categories with social class I representing professionals, social class II representing managerial and technical occupations, social class III representing subdivision into non-manual (III.N) and manual (III.M) skilled workers, social class IV representing partly skilled workers, and social class V representing unskilled manual workers. We re-categorized social class into manual (III.m-V) and non-manual (I-III.N) social classes [20]. Educational status (assessed at HLQ1) was based on the highest qualification attained and was categorized into 4 groups as follows: degree or equivalent, A-level or equivalent, O-level or equivalent, and less than O-level or no qualifications. O-level indicates educational attainment to the equivalent of completion of schooling to the age of 15 y, and A-level indicates educational attainment to the equivalent of the completion of schooling to the age of 17 y. Educational level was categorized into a binary variable: “at least O-level” (which includes O-level, A-level, and degree) versus “no qualifications” [20].

### Statistical analyses

All analyses were conducted using the Statistical Package for the Social Sciences for Windows version 20 (SPSS Inc, Chicago, IL, USA). A p-value < 0.05 was considered statistically significant.

We examined risk-factor distributions in men and women by 25(OH)D category. Potential confounders such as, age, BMI, social class, educational level, calcium intake, alcohol intake, smoking status, menopausal status, HRT use, supplement use, month of blood drawn, history of diabetes, cancer, osteoporosis, arthritis and previous fractures were evaluated for inclusion in the Cox regression models. Concentrations of 25(OH)D showed marked seasonal variations, and thus, all results were adjusted for the month of assessment as well as for age. The direction of the risk factors between men and women were similar and thus, analyses were performed for men and women together.

Cox proportional hazard models were used to determine hazard ratios (HR) of fractures for the two main exposures (A) by categories of plasma 25(OH)D with adjustment for: 1) age, sex and month of blood sampling, 2) model 1 and BMI, smoking status, alcohol intake, supplement use and history of fractures and 3) Model 2 and physical activity and (B) by physical activity where model 1 excluded month of blood sampling.

We tested the potential interaction between physical activity levels (active vs. inactive) and categories of 25(OH)D serum concentrations and between age (< = 65 y vs. >65 y) and 25(OH)D serum concentrations by including these as an additional model (model 4) and using the likelihood ratio test (LRT) to test for significance compared to model 3.

We also estimated HRs for fracture incidence up to 2, 4, 6, 8, 10 and 12-years of follow-up, per 20-nmol/L increase in serum 25(OH)D using model 1, 2 and 3.

## Results

After 15 y of follow-up, 1183 of the 14,624 participants (8.1%) had a fracture on any site; the rate among women (103/1000) was 2 times higher than among men (53/1000). Hip fracture was the most common fracture among adults older than 65 and other fractures apart from wrist, lumbar spine and hip were the most common ones among adults younger than 65 (S1 Fig). Among the men 39% and among women 45%, had a 25(OH)D concentration below 50 nmol/L; 28% and 24% respectively had a concentration above 70 nmol/L.

Characteristics of participants by 25(OH)D category measured in 1997–2000 are shown in Table 1. Serum 25(OH)D were inversely related to age, BMI, alcohol intake, smoking status, supplement use and physical inactivity in men and women, while additionally menopausal status, HRT use, history of arthritis and previous fractures were inversely related among women only (p< 0.05).

**Table 1:** Descriptive characteristics of 14624 subjects in the EPIC-Norfolk 1997–2000 by serum 25 (OH)D [total of and 25(OH)D2 and 25(OH)D3] category1.

	Total	Plasma 25 (OH)D category (nmol/L)										*P* (ANOVA)
		<30		30 to <50		50 to <70		70 to <90		≥90		
		*n*		*n*		*n*		*n*		*n*		
Men												
Total 25(OH)D (nmol/L)^1^	6485	603	23.3 ± 4.8^2^	1952	40.9 ± 5.7	2135	59.5 ± 5.6	1242	78.5 ± 5.6	553	105.5 ± 15.0	**<0.001**
25(OH)D_3_ (nmol/L)	6485	603	23.1 ±4.9	1952	40.7 ± 5.9	2135	59.2 ± 5.9	1242	78.3 ± 5.8	553	104.8 ± 15.9	**<0.001**
25(OH)D_2_ (nmol/L)	359	20	4.7 ± 1.8	108	5.0 ± 3.6	111	5.7 ± 3.7	70	5.1 ± 2.7	50	8.5 ± 15.8	**0.028**
Age (y)	6485	603	63.3 ± 9.3	1952	63.2 ± 9.0	2135	63.0 ± 9.1	1242	62.4 ± 8.8	553	61.7 ± 8.7	**0.001**
BMI (kg/m^2^)	6474	599	27.3 ± 3.8	1948	27.2 ± 3.5	2133	26.9 ± 3.2	1241	26.5 ± 3.1	553	26.2 ± 2.9	**<0.001**
Alcohol (units/wk)	6393	592	10.4 ± 12.8	1918	9.7 ± 11.3	2105	9.6 ± 11.1	1229	10.2 ± 11.4	549	12.0 ± 12.3	**<0.001**
Calcium intake (mg/d)^4^	4997	447	988.7 ± 307.5	1482	1018.9 ± 295.9	1668	1017.2 ± 292.2	943	1019.7 ± 292.4	457	976.7 ± 299.9	0.74
*Social class*												0.21
Non-manual	61.8 (3960)^3^	65.8 (389)		62.1 (1199)		61.8 (1302)		60.1 (741)		60.6 (329)		
Manual	38.2 (2444)	34.2 (202)		37.9 (731)		38.2 (806)		39.9 (491)		39.4 (214)		
*Educational level*												0.65
No qualification	27.4 (1778)	25.0 (151)		27.4 (535)		27.9 (597)		28.0 (349)		26.4 (146)		
O-level or higher qualification	72.6 (4721)	75.0 (452)		72.6 (1421)		72.1 (1544)		72.0 (896)		73.6 (408)		
Supplement users												**<0.001**
Non-supplement	74.4 (4818)	83.8 (503)		77.3 (1509)		73.3 (1566)		69.0 (855)		69.6 (385)		
Supplement	25.6 (1661)	16.2 (97)		22.7 (442)		26.7 (569)		31.0 (385)		30.4 (168)		
*Current smokers*												**<0.001**
Current	8.1 (521)	15.2 (91)		8.4 (163)		6.6 (139)		6.6 (81)		8.6 (47)		
Former	56.2 (3616)	52.3 (314)		56.3 (1091)		56.2 (1189)		57.4 (709)		57.0 (313)		
Never	35.8 (2302)	32.5 (195)		35.3 (684)		37.3 (789)		36.0 (445)		34.4 (189)		
History of diabetes	4.6 (275)	5.7 (32)		5.4 (99)		4.1 (80)		3.7 (43)		4.1 (21)		0.08
History of cancer	5.8 (346)	6.0 (33)		5.8 (104)		5.7 (110)		5.8 (67)		6.3 (32)		0.99
History of osteoporosis	1.5 (88)	1.3 (7)		1.2 (21)		1.5 (30)		2.2 (25)		1.0 (5)		0.20
History of arthritis	26.3 (1588)	22.7 (126)		25.3 (458)		26.4 (522)		28.4 (332)		29.0 (150)		0.06
History of previous fractures	8.0 (521)	8.3 (50)		8.0 (157)		8.0 (170)		7.9 (98)		8.3 (46)		0.99
*Physical activity*												**<0.001**
Inactive	27.2 (1766)	36.3 (219)		29.6 (578)		25.7 (549)		24.2 (300)		21.7 (120)		
Moderately inactive	24.9 (1618)	27.4 (165)		25.9 (505)		24.1 (514)		24.6 (305)		23.3 (129)		
Moderately active	25.0 (1623)	21.1 (127)		24.5 (478)		25.7 (548)		26.2 (325)		26.2 (145)		
Active	22.8 (1478)	15.3 (92)		20.0 (391)		24.5 (524)		25.1 (312)		28.8 (159)		
Women												
Total 25(OH)D (nmol/L)^1^	8139	1039	23.2 ± 5.0	2591	40.6 ± 5.7	2557	59.4 ± 5.7	1334	78.7 ± 5.6	618	104.4 ± 14.7	**<0.001**
25(OH)D_3_ (nmol/L)	8139	1039	23.0 ± 5.1	2591	40.2 ± 5.9	2557	59.1 ± 5.9	1334	78.2 ± 6.1	618	103.9 ± 14.8	**<0.001**
25(OH)D_2_ (nmol/L)	564	47	4.3 ± 1.3	169	5.5 ± 4.3	182	5.4 ± 4.1	109	6.0 ± 4.6	57	5.3 ± 5.6	0.25
Age (y)	8139	1039	62.9 ± 9.5	2591	62.3 ± 9.3	2557	61.3 ± 8.8	1334	60.3 ± 8.7	618	59.0 ± 8.1	**<0.001**
BMI (kg/m^2^)	8127	1036	27.3 ± 5.0	1586	26.9 ± 4.6	2553	26.4 ± 4.1	1334	25.8 ± 3.8	618	25.2 ± 3.7	**<0.001**
Alcohol (units/wk)	7983	1017	4.2 ± 5.4	2533	4.2 ± 5.3	2521	4.7 ± 5.9	1305	5.2 ± 5.8	607	5.6 ± 6.0	**<0.001**
Calcium intake (mg/d) ^4^	7044	804	942.5 ± 275.5	2049	963.4 ± 282.8	2069	974.5 ± 277.8	1077	961.5 ± 284.0	508	922.1 ± 284.7	0.76
*Menopausal status*												**0.005**
Premenopausal	5.9 (463)	6.2 (62)		5.6 (140)		5.4 (133)		7.4 (95)		5.6 (33)		
Early perimenopausal	3.3 (258)	2.2 (22)		3.6 (90)		3.4 (85)		3.2 (41)		3.4 (20)		
Late perimenopausal	18.1 (1422)	16.2 (163)		15.9 (399)		18.6 (459)		19.7 (254)		25.0 (147)		
Postmenopausal	72.8 (5722)	75.5 (761)		75.0 (1884)		72.6 (1791)		69.7 (899)		65.9 (387)		
HRT use												**<0.001**
Current	21.3 (1729)	17.3 (179)		19.2 (496)		20.5 (524)		25.6 (341)		30.6 (189)		
Former	17.4 (1412)	14.7 (152)		17.2 (444)		18.6 (475)		17.3 (230)		18.0 (111)		
Never	61.3 (4985)	68.1 (706)		63.7 (1647)		60.9 (1554)		57.1 (761)		51.4 (317)		
*Social class*												0.55
Non-manual	64.0 (5130)	62.5 (633)		64.7 (1652)		63.4 (1594)		65.3 (865)		63.2 (386)		
Manual	36.0 (2888)	37.5 (380)		35.3 (902)		36.6 (922)		34.7 (459)		36.8 (225)		
*Educational level*												0.68
No qualification	37.5 (3069)	38.1 (397)		37.3 (969)		38.4 (985)		37.0 (497)		35.5 (221)		
O-level or higher qualification	62.5 (5105)	61.9 (645)		62.7 (1632)		61.6 (1580)		63.0 (847)		64.5 (401)		
Supplement users												**<0.001**
Non-supplement	59.9 (4869)	75.1 (780)		64.6 (1673)		55.4 (1415)		50.7 (676)		52.7 (325)		
Supplement	40.1 (3265)	24.9 (259)		35.4 (916)		44.6 (1141)		49.3 (657)		47.3 (292)		
*Current smokers*												**<0.001**
Current	8.1 (654)	11.9 (122)		8.0 (204)		7.1 (181)		6.4 (85)		10.1 (62)		
Former	32.9 (2656)	31.4 (322)		32.5 (835)		33.5 (850)		32.0 (424)		36.8 (225)		
Never	59.0 (4758)	56.8 (583)		59.5 (1527)		59.4 (1508)		61.6 (816)		53.0 (324)		
History of diabetes	2.7 (193)	3.6 (32)		3.0 (66)		2.4 (54)		2.4 (29)		2.1 (12)		0.26
History of cancer	9.9 (692)	10.9 (95)		10.3 (227)		9.3 (203)		9.4 (110)		10.4 (57)		0.62
History of osteoporosis	5.3 (373)	5.1 (45)		6.2 (136)		5.3 (117)		4.3 (51)		4.4 (24)		0.15
History of arthritis	37.4 (2770)	39.6 (371)		40.3 (941)		36.6 (847)		34.2 (424)		32.7 (187)		**<0.001**
History of previous fractures												
Physical activity												**<0.001**
Inactive	26.1 (2127)	36.0 (374)		28.5 (739)		23.9 (610)		21.4 (286)		19.1 (118)		
Moderately inactive	32.8 (2666)	31.5 (327)		33.1 (857)		33.4 (853)		33.4 (445)		29.8 (184)		
Moderately active	23.9 (1946)	19.6 (204)		23.4 (607)		25.7 (657)		24.0 (320)		25.6 (158)		
Active	17.2 (1400)	12.9 (134)		15.0 (388)		17.1 (437)		21.2 (283)		25.6 (158)		

^1^Total 25(OH)D comprises the sum of 25(OH)D2 AND 25(OH)D3. EPIC-Norfolk, European Prospective Investigation into Cancer and Nutrition-Norfolk; 25(OH)D, 25-hidroxyvitamin D; HRT, hormone replacement therapy

^2^Mean ± SD (all such values)

^3^percentage (n)

^4^Calcium intake adjusted for energy intake

HRs for total fractures by 25(OH)D category are shown in Table 2. We observed a significant 19% lower fracture risk among those individuals categorized with 25(OH)D of 50 to <70 nmol/L compared to individuals with concentrations <30 nmol/L when adjusted for model 2. This association only marginally attenuated after including physical activity in the model. Further adjustment for menopausal status among women did not materially change our observations (results not shown). No further significant associations were observed for the other 25(OH)D categories. A modest J-shape relationship with fracture risk and 25(OH)D was observed.

**Table 2:** Rates and HRs by serum 25(OH)D category for fractures in 14242 men and women in the EPIC-Norfolk 1997–2015*.

	Serum 25(OH)D category (nmol/L)				
	<30	30 to <50	50 to <70	70 to <90	>90
% (n)	10.2 (167)	8.9 (403)	7.3 (344)	7.5 (192)	6.6 (77)
HR (95% CI)^1^	1	0.93 (0.78, 1.12)	0.84 (0.70, 1.02)	0.93 (0.75, 1.16)	0.92 (0.69, 1.21)
HR (95% CI)^2^	1	0.91 (0.76, 1.10)	**0.81 (0.67, 0.99)**	0.93 (0.75, 1.17)	0.86 (0.65, 1.15)
HR (95% CI)^3^	1	0.91 (0.76, 1.10)	**0.82 (0.67, 0.99)**	0.93 (0.75, 1.17)	0.86 (0.65, 1.14)

*Data for those with complete case analysis

^1^Age, sex and month adjusted (model 1)

^2^Age, sex, month, BMI, smoking, alcohol, supplement use and history of fractures adjusted (model 2)

^3^Age, sex, month, BMI, smoking, alcohol, supplement use, history of fractures and physical activity adjusted (model 3)

HRs for total fractures by physical activity are included in S1 Table. No statistically significant associations were found for fracture risk by physical activity level. HRs for total fractures by age are included in S2 Table. For younger adults with the highest 25(OH)D category we observed a 40% lower risk of fractures compared with those in the lowest 25(OH)D category (HR: 0.60; 95%CI: 0.36–0.99). For older participants we observed a J-shaped association with fracture risk when the 3^rd^ 25(OH)D category was used as the reference category (data not shown).

**Fig 1:**
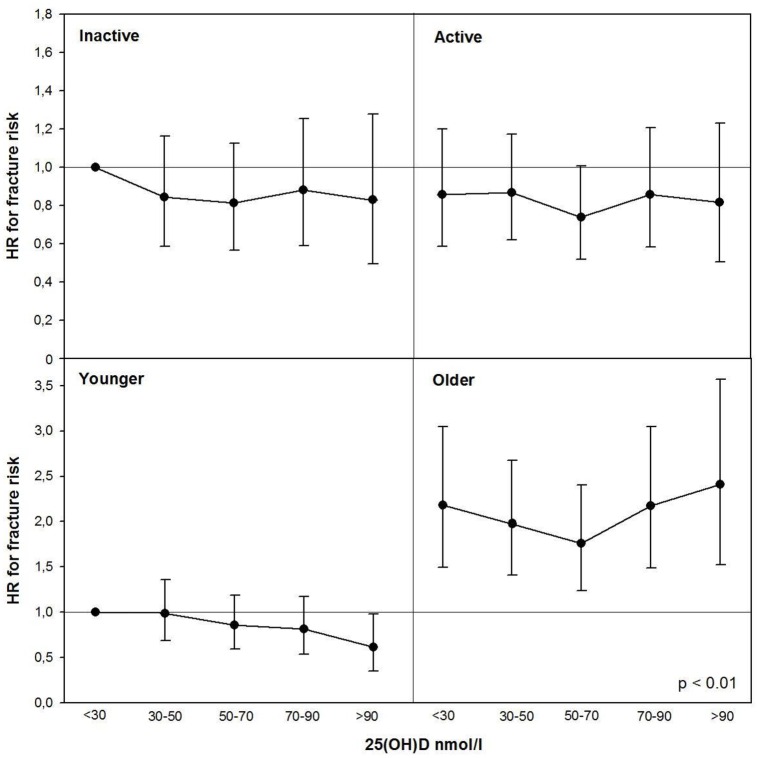
HRs for fracture risk by physical activity- and age-25(OH)D interactions by Cox proportional hazard model after adjustment using the model 4.

Interaction testing between physical activity and 25(OH)D and age and 25(OH)D were also formally tested (Fig 1). No significant interaction was observed between physical activity and 25(OH)D (P_LRT_ = 0.782), but age modified the association between 25(OH)D and fracture risk (P_LRT_ < 0.01). Among younger participants higher 25(OH)D concentrations were associated with lower risk; whereas among older participants higher 25(OH)D concentrations were associated with higher fracture risk.

HRs for fracture incidence by 2 year increments of follow-up time are shown in Table 3. A significant association was observed for fracture risk at 8 and 10 years of follow-up per 20 nmol/L increase. No linear associations were found at the end of follow-up conform our results in Table 2.

**Table 3:** HRs for fracture incidence by follow-up time per 20-nmol/L increase in serum 25(OH)D in 14242 men and women in the EPIC-Norfolk 1997–2015.

Follow-up time	No. of events	HR (95% CI)	P
2 y			
Age, sex, and month adjusted	76	0.84 (0.67, 1.05)	0.12
Multivariable adjusted^1^		0.85 (0.67, 1.07)	0.17
Multivariable adjusted^2^		0.83 (0.66, 1.05)	0.12
4 y			
Age, sex, and month adjusted	172	0.88 (0.76, 1.02)	0.10
Multivariable adjusted^1^		0.88 (0.76, 1.03)	0.10
Multivariable adjusted^2^		0.88 (0.75, 1.02)	0.09
6 y			
Age, sex, and month adjusted	288	0.90 (0.81, 1.01)	0.08
Multivariable adjusted^1^		0.92 (0.82, 1.03)	0.15
Multivariable adjusted^2^		0.92 (0.82, 1.03)	0.14
8 y			
Age, sex, and month adjusted	447	0.89 (0.81, 0.97)	**0.009**
Multivariable adjusted^1^		0.89 (0.81, 0.98)	**0.016**
Multivariable adjusted^2^		0.89 (0.81, 0.98)	**0.016**
10 y			
Age, sex, and month adjusted	597	0.90 (0.83, 0.98)	**0.011**
Multivariable adjusted^1^		0.92 (0.84, 0.99)	**0.031**
Multivariable adjusted^2^		0.92 (0.85, 0.99)	**0.036**
12 y			
Age, sex, and month adjusted	783	0.94 (0.88, 1.01)	0.08
Multivariable adjusted^1^		0.95 (0.89, 1.02)	0.14
Multivariable adjusted^2^		0.95 (0.89, 1.02)	0.16
Final			
Age, sex, and month adjusted	1183	0.97 (0.92, 1.03)	0.32
Multivariable adjusted^1^		0.97 (0.92, 1.03)	0.37
Multivariable adjusted^2^		0.97 (0.92, 1.03)	0.35

^1^Adjusted for age, sex, month, BMI, smoking, alcohol supplement use, and history of fractures (model 2)

^2^Adjusted for age, sex, month, BMI, smoking, alcohol, supplement use, history of fractures and physical activity (model 3)

## Discussion

This is a large cohort of middle aged and older men and women examining the role of physical activity and 25(OH)D serum concentrations separately and their potential for interaction with fracture risk. Results have shown that 25(OH)D concentrations of 50–70 nmol/l measured between 1997 and 2000 were inversely associated with fracture risk after 15 years of follow up. In older adults this association was observed to be J-shaped; whereas, among younger adults, the association was observed to be linearly inverse. No association was observed between physical activity and fracture risk.

The dose-response relation between serum 25(OH)D concentrations and bone health and the nature of the relationship, whether threshold or U-shaped is currently under debate [16, 21]. The Food and Nutrition Board (FNB) at the Institute of Medicine (IOM) of the National Academies concluded in 2010 that serum 25(OH)D levels above 125–150 nmol/L should be avoided, as even lower serum levels (approximately 75–120 nmol/L) are associated with increases in all-cause mortality and higher incidence of falls and fractures among the elderly [16], however a recent meta-analysis of all-cause mortality concluded that the relation between 25(OH)D concentration and all-cause mortality is flat above 90nmol/L [22]. The prospective population-based CHAMP Study found that, after a mean of 4.3 years of follow-up, the relationship between baseline 25(OH)D and fracture risk in men was U-shaped with risk of fracture being greater at concentrations <36 nmol/L and ≥72 nmol/L compared to ≥60 to ≤72 nmol/L [21]. A large study of women aged ≥ 69 years followed for an average of 4.5 years observed that both lower (<50 nmol/L) and higher (≥75 nmol/L) 25(OH)D concentrations at baseline to be associated with a greater risk of bone frailty [23]. We did not observe any significant association when we combined younger and older adults. This trend was significantly confirmed in older adults when performing the age and vitamin D interaction testing. Previous studies have reported associations between high 25(OH)D levels and fracture risk [23].

Our results are surprising compared with our findings in 2013 when we reported results from this cohort over a 9 year follow up period (until March 2009) [4]. Those individuals at higher 25(OH)D categories (30 to <50, 50 to <70, 70 to <90 and ≥90 nmol/L) compared with those at the lowest category (<30 nmol/L) were at lower risk for fractures (with significant linear trends) [4]. In the current analysis (end of follow-up 2015, an average of 15 ± 2.3 y of follow-up) fracture events doubled (563 vs.1183), but the associations between fracture risk and 25(OH)D were only significant in younger adults for concentrations > = 90 nmol/L. The length of follow-up time may be an issue, as a single blood sample may not represent usual 25(OH)D status over a long period of follow up [6]. It is plausible that in the intervening years, many individuals may have started taking vitamin D supplements due to a change in public health and clinical recommendations [24] and therefore changing their exposure status (resulting in a potential shift upwards for serum 25(OH)D, particularly among the over 65 years old, to whom the policies applied).

In 2010, the Community-Based Cohort of Elderly Men, in Sweden with a follow-up greater than 20 years did not observe associations between 25(OH)D serum concentrations and fracture risk [25]. They concluded that genetic adaptations to limited UV light may explain this finding, although we hypothesize that the time between exposure assessment and end of follow-up could partially explain these results.

The biological mechanism linking 25(OH)D deficiency with fracture is still unclear. Prolonged vitamin D insufficiency in the elderly is associated with reductions in both bone mineral density (BMD) and muscle mass which may lead to an increased falls risk and consequently to increased fracture rates as 90% of fractures in the elderly occur after a fall [26]. Besides, vitamin D deficiency may be closely associated with the production of abnormal levels of calcium-phosphorus, consequently resulting in diminished collagen matrix mineralization [27].

Prolonged immobilization is a risk factor for bone and muscle mass loss, and consequently, future fracture [28, 29]. Observational studies have suggested that the age-related decline in BMD is attenuated, and the relative risk of fractures is reduced, in people who are physically active, even when the activity is not particularly vigorous [9, 30]. However, we failed to find associations between physical activity and fracture risk separately and across categories of 25(OH)D serum concentrations. Given the current state of knowledge from multiple randomized controlled trials, weight-bearing endurance activities, such as stair climbing or jogging, activities that involve jumping like dancing, and resistance exercise like weight lifting are recommended to preserve bone health in adulthood [30, 31]. Thus, reviews and meta-analyses of randomized trials suggest that balance and flexibility training effectively reduce risk of falling in older adults [32, 33].

It is also important to clarify that benefits of exercise in middle-aged and older people may be reflected by attenuation in the rate of bone loss, rather than an increase in bone mass. In this regard, the peak bone mass attained before the end of the third decade could be crucial to avoid fractures in the elderly [30].

Little is known about the combined effects of vitamin D and exercise. Results from randomized control trials have suggested that vitamin D could enhance strength, balance and mobility, and consequently reduce the risk of falls and fractures [11, 12]. When we studied the relationship between physical activity level and fracture risk across categories of 25(OH)D we did not observe a linear association nor interaction. The latter suggests that vitamin D status was an independent predictor of fracture risk or that both exposures have changed over time and one baseline measurement is not enough to detect any possible association or interaction.

### Strengths and limitations

The present study has several limitations as well as strengths. Regarding limitations, firstly the physical activity index used was a combined index of leisure time and occupational activity. While this index has been objectively validated against heart rate measurements, and has demonstrated utility in being a strong predictor of mortality and cardiovascular disease [34], this index did not take into account the type of exercise undertaken (e.g. weight bearing, high impact) and this could partially explain why we did not find associations between our measure of physical activity and fracture risk [35]. Also, those individuals who exercise outdoors may get additional benefits from sun exposure than those who exercise indoors [36].

Some have suggested that while increasing physical activity may improve bone health, it may increase fracture risk by increasing falls [37]. Lastly, it must be recognized that the opportunity for falling probably increases as people become more physically active, particularly in community dwelling elderly [38].

There is some evidence suggesting that physical activity habits during childhood may have long-lasting benefits on bone health [39]. Unfortunately, we did not have information on physical activity when peak bone mass was attained in this cohort, and therefore we could not investigate the relationship between physical activity in early adulthood, adolescence or childhood with fracture risk.

Misclassification of physical activity levels due to under- or over-reporting is inevitable, although the physical activity questionnaire has shown good reliability in classifying individuals into physical activity levels [18]. In contrast, we did not keep track of physical activity level over time using similar assessment methods and those individuals who were physically active when data were collected could have decreased their activity and consequently being more prone to fractures as has been suggested [9].

Unfortunately, we did not keep track of the place while exercising if indoors or outdoors. Moreover, the causes of fractures, whether by fragility or high impacts, were not registered and this could have explained why we did not observe any interaction between physical activity and 25(OH)D, because fractures could be due to high impacts while exercising.

As previously discussed, a single baseline blood sample to assess vitamin D status may not have adequately characterised an individual’s exposure over a long follow up time period, especially over an era of changing clinical and public health practice in vitamin D supplementation, particularly to groups perceived as most vulnerable to fractures such as older women [40]. Moreover, while ELISA assay for 25(OH)D_3_ are generally accepted the LC/MS methods would be more precise, as it allows detection and measurement of other recently described mono-xydroxymetabolites that are biologically active [41–44].

Potential strengths of our study are the large population sample including both men and women, large pool of fractures (1183 cases), long duration of follow-up and detailed assessment of potential confounders.

## Conclusions

Although physical activity (separately and in combination with vitamin D) was not related to fracture risk in this large-sample cohort study, the interpretation of findings are limited by the potential measurement error in the use of a single baseline physical activity assessment. Vitamin D status appears inversely related to fractures in middle aged adults. In older adults, the dose response relationship between baseline vitamin D status and fracture risk appeared to be J-shaped. Changes in clinical and public health practice regarding vitamin D supplementation may partially explain this finding.

## Supporting Information

S1 Fig Fracture sites stratified by gender and age. (TIF)

S1 Table Rates and HRs by serum 25(OH)D category and physical activity for fractures in 13031 men and women in the EPIC-Norfolk 1997–2015. ^1^Age, sex and month adjusted. ^2^Age, sex, month, BMI, supplement use, smoking, alcohol, history of fractures adjusted.
(DOCX)

S2 Table Rates and HRs by serum 25(OH)D and age categories for fractures in 13031 men and women in the EPIC-Norfolk 1997–2015. ^1^Sex and month adjusted. ^2^Sex, month, BMI, supplement use, smoking, alcohol, history of fractures adjusted.
(DOCX)
