# Supplementary figures and images for: Fracture Risk in Relation to Serum 25-Hydroxyvitamin D and Physical Activity: Results from the EPIC-Norfolk Cohort Study
Source: PLoS One. 2016 Oct 17;11(10):e0164160. doi: 10.1371/journal.pone.0164160 (PMC5066971; doi:10.1371/journal.pone.0164160)

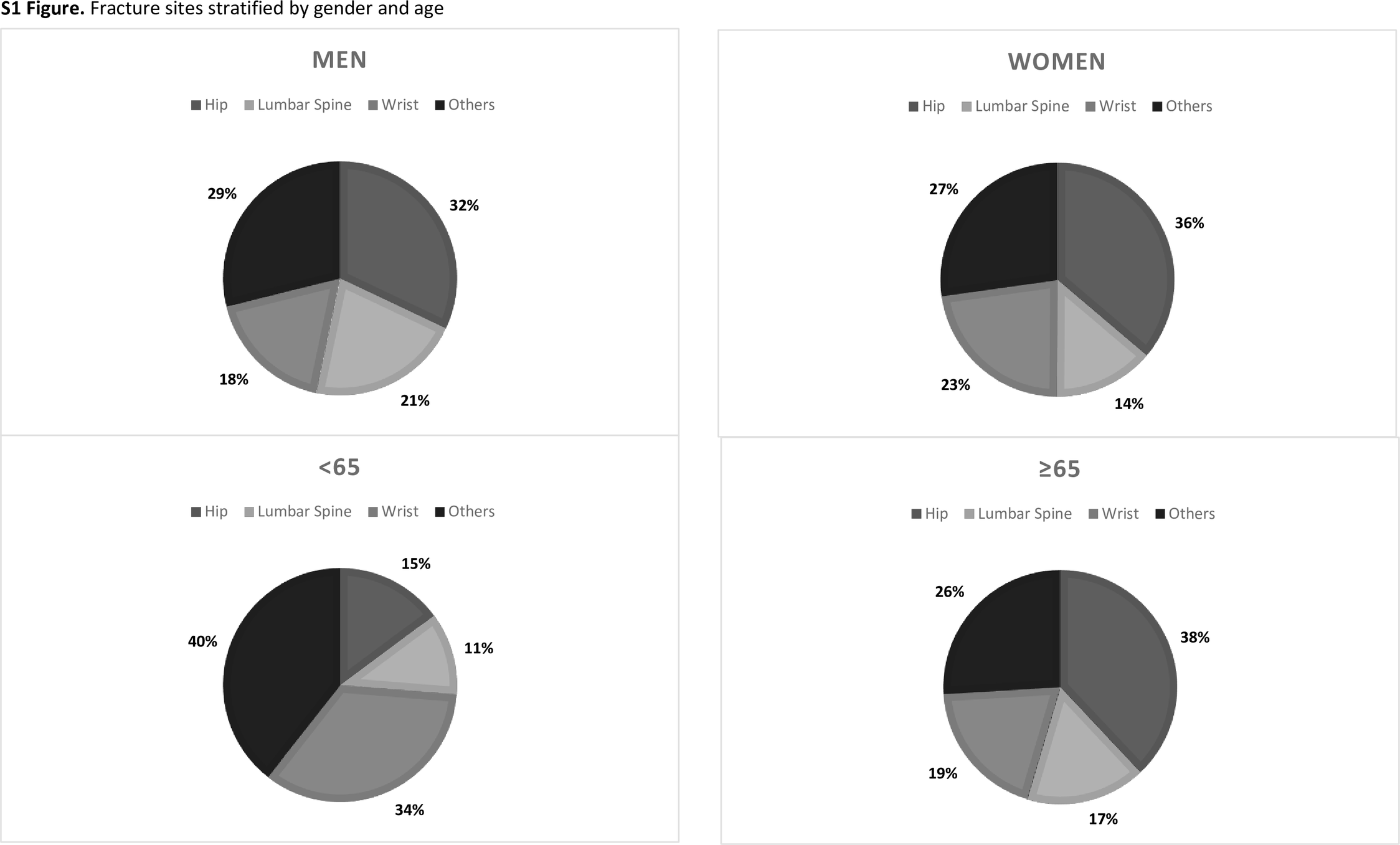

Supplement: S1 Fig — (TIF) [file pone.0164160.s001.tif]
