# Supplementary material for: Fracture Risk in Relation to Serum 25-Hydroxyvitamin D and Physical Activity: Results from the EPIC-Norfolk Cohort Study
Source: PLoS One. 2016 Oct 17;11(10):e0164160. doi: 10.1371/journal.pone.0164160 (PMC5066971; doi:10.1371/journal.pone.0164160)
Supplement: S1 Table — 1Age, sex and month adjusted. 2Age, sex, month, BMI, supplement use, smoking, alcohol, history of fractures adjusted. (DOCX) [file pone.0164160.s002.docx]

| **S1 Table. Rates and HRs by serum 25(OH)D category and physical activity for fractures in 13031 men and women in the EPIC-Norfolk 1997-2015*** | | | | | | |
| --- | --- | --- | --- | --- | --- | --- |
|  |  | Serum 25(OH)D category (nmol/L) | | | | |
|  |  | <30 | 30 to <50 | 50 to <70 | 70 to <90 | ≥90 |
| Inactive | |  |  |  |  |  |
|  | % (n) | 9.2 (51) | 7.2 (135) | 6.6 (142) | 6.7 (83) | 5.6 (35) |
|  | HR (95% CI)^1^ | 1 | 0.79 (0.56, 1.10) | 0.73 (0.52, 1.02) | 0.77 (0.53, 1.12) | 0.70 (0.44, 1.11) |
|  | HR (95% CI)^2^ | 1 | 0.85 (0.60, 1.19) | 0.81 (0.57, 1.14) | 0.87 (0.59, 1.28) | 0.83 (0.52, 1.32) |
| Active | |  |  |  |  |  |
|  | % (n) | 10.7 (116) | 10.0 (268) | 8.0 (202) | 8.2 (109) | 7.6 (42) |
|  | HR (95% CI)^1^ | 1 | 1.09 (0.85, 1.39) | 0.95 (0.73, 1.22) | 1.02 (0.76, 1.37) | 1.14 (0.78, 1.67) |
|  | HR (95% CI)^2^ | 1 | 1.03 (0.81, 1.32) | 0.88 (0.68, 1.15) | 1.02 (0.75, 1.38) | 0.99 (0.68, 1.46) |
| *Data for those with complete case analysis | | | | | | |
| ^1^Age, sex and month adjusted | | | | | | |
| ^2^Age, sex, month, BMI, supplement use, smoking, alcohol, history of fractures adjusted | | | | | | |
