# Supplementary material for: Fracture Risk in Relation to Serum 25-Hydroxyvitamin D and Physical Activity: Results from the EPIC-Norfolk Cohort Study
Source: PLoS One. 2016 Oct 17;11(10):e0164160. doi: 10.1371/journal.pone.0164160 (PMC5066971; doi:10.1371/journal.pone.0164160)
Supplement: S2 Table — 1Sex and month adjusted. 2Sex, month, BMI, supplement use, smoking, alcohol, history of fractures adjusted. (DOCX) [file pone.0164160.s003.docx]

| **S2 Table. Rates and HRs by serum 25(OH)D and age categories for fractures in 13031 men and women in the EPIC-Norfolk 1997-2015*** | | | | | | |
| --- | --- | --- | --- | --- | --- | --- |
|  |  | Serum 25(OH)D category (nmol/L) | | | | |
|  |  | <30 | 30 to <50 | 50 to <70 | 70 to <90 | ≥90 |
| Younger adults | |  |  |  |  |  |
|  | % (n) | 5.8 (55) | 5.2 (139) | 4.8 (139) | 4.4 (74) | 3.3 (28) |
|  | HR (95% CI)^1^ | 1 | 0.98 (0.70, 1.36) | 0.86 (0.61, 1.21) | 0.76 (0.52, 1.12) | **0.57 (0.35, 0.94)** |
|  | HR (95% CI)^2^ | 1 | 0.96 (0.69, 1.34) | 0.80 (0.57, 1.13) | 0.79 (0.53, 1.17) | **0.60 (0.36, 0.99)** |
| Older adults | |  |  |  |  |  |
|  | % (n) | 16.1 (112) | 14.1 (264) | 11.6 (205) | 13.4 (118) | 15.1 (49) |
|  | HR (95% CI)^1^ | 1 | 0.94 (0.74, 1.20) | 0.83 (0.64, 1.07) | 0.97 (0.73, 1.29) | 1.26 (0.88, 1.80) |
|  | HR (95% CI)^2^ | 1 | 0.91 (0.72, 1.17) | 0.83 (0.64, 1.07) | 1.01 (0.75, 1.36) | 1.16 (0.80, 1.67) |
| *Data for those with complete case analysis | | | | | | |
| ^1S^Sex and month adjusted | | | | | | |
| ^2^Sex, month, BMI, supplement use, smoking, alcohol, history of fractures adjusted | | | | | | |
